# Proximate Composition, Amino Acid Profile, and Mineral Content of Four Sheep Meats Reared Extensively in Morocco: A Comparative Study

**DOI:** 10.1155/2021/6633774

**Published:** 2021-01-20

**Authors:** Kamal Belhaj, Farid Mansouri, Abdessamad Ben moumen, Marianne Sindic, Marie-Laure Fauconnier, Mohamed Boukharta, Hana Serghini Caid, Ahmed Elamrani

**Affiliations:** ^1^Laboratory of Agricultural Production Improvement, Biotechnology and Environment, Faculty of Sciences, University Mohammed First, 717, Oujda 60000, Morocco; ^2^Analysis Quality and Risk Unit, Laboratory of Food Quality and Safety (QSPA), Gembloux Agro-Bio Tech, University of Liège, Gembloux 5030, Belgium; ^3^General and Organic Chemistry Unit (CGO), Gembloux Agro Bio-Tech, University of Liège, Gembloux 5030, Belgium; ^4^Institute of Agricultural Industries, High School of Charlemagne, Huy 4500, Belgium

## Abstract

This study aimed to compare the organoleptic and nutritional quality of four sheep meats produced in Morocco. This comparison was carried out by analyzing the proximate composition, amino acid profile, and mineral content of meat. The majority of the evaluated parameters were influenced by genetic and geographical factors (*p* < 0.05). The *longissimus lumborum* muscle had higher *a*^*∗*^ value in Timahdite and Ouled-Djellal breeds. The highest values of macroelement were recorded in samples of Beni-Guil meat sampled in the Tendrera region (BGT; 1067.3 mg/100 g), while the highest microelement content was registered in Timahdite meat (5.7 mg/100 g). Iron and zinc were the major identified trace elements, while phosphorus and potassium were the most abundant macroelements. The abundant amino acid was glutamic, while cysteine and methionine were least abundant. The higher essential amino acids index (IEAA) was recorded in the Timahdite sheep meat (162.20, 158.71, 161.02, and 159.65, respectively, for Timahdite, BGT, Ouled-Djellal breeds, and Beni-Guil meat sampled in Ain Beni Mathar region). From a nutritional point of view, the studied meats had a good protein and mineral quality, due to their richness in essential amino acids and microelements. The present study provided new insights on the organoleptic quality and the nutritional value of three Moroccan sheep meats reared in outdoor production system.

## 1. Introduction

Consumers often consider meat quality based on organoleptic properties and microbiological quality. However, meat quality can be evaluated in a more unbiased way by evaluating its chemical composition and physical proprieties. Ruminant's meat applies a crucial role in human nutrition. Besides, it represents an important constituent of a balanced and healthy diet due to its essential components such as amino acids, long-chain n-3 polyunsaturated fatty acids, and microelements, namely, iron, selenium, zinc, and copper. The different quality traits of red meat are influenced by several factors, that is, genetic, environmental and management factors. The meat composition varies from breeds, from animals, and from one muscle to another in the same animal [1]. In Morocco, breeding of small ruminants is considerably important due to its predominance throughout the kingdom. Generally, sheep are encountered in all regions and provinces of the kingdom, thanks to not only its adaptation to the majority of existing agroecosystems but also the biodiversity of this species. Sheep livestock in Morocco is generally destined for meat production where the majority of the males are intended for the feast of the sacrifice, and about 25% of female lambs are reserved to replace the elderly ewes. The rest are fattened and intended for slaughter to supply the red meat markets throughout the year. The Beni-Guil and “Daghma” breed are the main sheep breeds in Morocco. It is local and occupies the pasture of the eastern highlands of Morocco, in juxtaposition with the geographical livestock area of the Timahdite breed or “Bergui” indigenous of the Middle Atlas. These two sheep breeds are characterized by their rusticity, their cross ability, and their effective representation with a percentage of 12.58% and 9.8% of the Moroccan herd, respectively [2, 3]. Other nonautochthonous and unlabeled breeds are making their place into the sheep breeding landscape of Morocco. For example, the Ouled-Djellal breed, renowned for its weight characteristics, recently begins to find its own place in the oriental region of Morocco. These two breeds, Timahdite and Ouled-Djellal, are considered competitors of the Beni-Guil breed. In fact, Timahdite breed is considered as a competitive breed at national level and Ouled-Djellal as a competitor at a regional level including the highlands of the eastern region in relation to transhumance and its Algerian origin [4].

Currently, there is a strong trend towards to organic food and the consumption of lean meats from goats and sheep that reared on pasture. This conjuncture places the local breeds in a favorable position to the breeds selected for intensive breeding allowing high fattening performance. This study focuses on two main topics: (i) to compare and evaluate the nutritional value of the Beni-Guil (BG) breed meat with those of Timahdite (Ti) and Ouled-Djellal (ODj) and (ii) to determine the effect of geographical area on meat nutritional quality of the Beni-Guil sheep breed.

## 2. Material and Methods

### 2.1. Animal

Forty-eight carcasses of female lambs from three sheep breeds reared in Moroccan pastures, two indigenous breeds (BG (*n* = 24) and Ti (*n* = 12)), and one no-indigenous breed of Algerian origin called ODj (*n* = 12), which live together with the Beni-Guil sheep in highland of eastern Morocco, were used in this research. To study the geographical area effect, the samples were taken of meat from the Beni-Guil breed in the two main cradles of this breed: in the region of Ain Beni Mathar (BGA), *n* = 12 and in that of Tendrara (BGT), *n* = 12. Ain Beni Mathar region in the north and that of Tendrera in the south were chosen with a decreasing bioclimatic gradient from north to south, ranging from semiarid to the lower-arid and pre-Saharan arid. Animals were aged between 6 and 8 months and weaned at the age of 3 months equivalent to “L” category according to the regulation of the European Commission Nı 823/98. The animals have been slaughtered at 33-37 kg of live weight, with an average-high fattening state, in accordance with the Community Scale of Grading Sheep Carcasses EUROP (Sagot & Pottier, 2011b), and the conformation ranged between fair (O) and good (R) class according to the EUROP grid (Sagot & Pottier, 2011a). These BG and ODj lambs belong to herds raised in the eastern Morocco highland, and the Ti lambs belong to herds raised in the rural commune of El-Hajeb (middle Atlas). The breeds used in this study were reared in the same production and rearing system. Animals in eastern Morocco were reared on natural highlands in an arid to a semiarid environment. The diet of the ewes was composed of natural pastures when available and the same supplementation of 150 to 250 g per day based on alfalfa hay and barley in drought, connecting periods, and in some physiological stages, such as at the time of preparation for breeding (flushing) and for lambing (steaming). The lambing season occurred mainly from September to December. Four weeks before parturition, ewes received barley supplementation to avoid abortions and to improve milk production and quality after lambing. Fifteen days after lambing, the lambs were vaccinated against enterotoxaemia. In the first two months of age, the lambs stayed in the sheepfold during the day, giving them a supplement based on barley and alfalfa hay, ad libitum. Then, in the third month of age, the lambs were raised with their mothers until weaning at the age of 3 months and grazed on natural grassland pastures with additional feeding based on barley (100–120 g) and hay. Before slaughtering, the lambs had a finishing phase of 45 days based on barley (1 to 1.5 kg). The lambs had free access to water and mineral supplement (lick block).

In the pastoral system adopted by breeders of eastern Morocco and Middle Atlas, the adopted feeding calendar was dominated by grazing that lasted up to 8–12 months/year depending on climatic conditions (rainfall).

- In the Eastern region of Morocco, animals moved south in winter and north in summer within the same area to graze on the Alfa (*Stipa tenacissima*) and wormwood *(Artemisia herba-alba*) steppes. In July-August, the animals fed on cereal stubbles. The dominant plant species in the steppe and highland pasture of Eastern Morocco included wormwood (*Artemisia herba-alba*), alfa grass (*Stipa tenacissima*), sparta grass (*Lygeum spartum*), Atriplex (*Atriplex halimus)*, ray-grass (*Lolium perenne* L.), laser white (*Laserpitium latifolium*), and sweet broom (*Arthrophytum scoparium*), along with the presence of other species, such as *Bromus* spp., *Eruca vesicaria* (roquette), *Stipa capensis,* and *Medicago* spp.
- In the Middle Atlas, the pair rangelands/forest was used throughout the year. In an altitude of 1.500 meters, breeders used stubble and crop residues and coproducts from market gardening in summer, fallow in spring, and raw barley in winter. This was the case of El-Hajeb region, where the samples of the Timahdite breed were taken. The dominant plant species in this geographical area were *Quercus rotundifolia*, *Stipa tenacissima*, *Dactylis glomerata*, *Festuca* spp., *Cynosurus elegans*, *Crataegus laciniata*, *Arrhenatherum elatius*, and *Juniperus phoenicea,* along with the presence of other species, such as *Avena* spp., *Papaver* spp., *Trifolium* spp., *Hordeum murinum,* and *Vicia* spp.

The sheep farmers were members of the National Association of Breed Producer (ANOC); they adopt a rhythm of lambing per year. Sampling was done with the assistance of the slaughterhouse veterinarian. Forty-eight samples of *longissimus lumborum* muscle (LLM) were taken 24 hours after slaughtering (12 animals for each breed).

### 2.2. Evaluation of Meat Quality

The ultimate pH was measured 24 hours postmortem in LL muscle using a pH meter with penetration probe (pH/Cond 340i WTW, Weilheim, Germany).

The meat color was estimated according to the CIELAB system proposed by the international center of lighting (CIE) using a chromameter (Konica Minolta CR400 (*L*^*∗*^ = Clarity, *a*^*∗*^ = Red-Green color, *b*^*∗*^ = Yellow-Blue color)). A D65 illuminant was used at an observation angle of 10° and with an aperture of 30 mm. The instrument was calibrated using white standard coordinates. Chromaticity [*C*^*∗*^=(*a*^*∗*^2^^+*b*^*∗*^2^^)^(1/2)^] and hue angle (*H*^*∗*^ = arctan (*b*^*∗*^/*a*^*∗*^)) were calculated [5].

The meat juiciness was estimated by two methods: by application of a mechanical force (water-holding capacity or expressible juice) and thermal (cooking loss: CL). To determine the loss of juice during cooking, firstly the meat was weighed and then placed in polyethylene bags in order to cook it in a water bath up to a core temperature of 75°C during one hour. After the cooking, the samples were cooled with cold water/30 minutes, and then the meat was removed from the bags, dried with paper towels, and weighed again. CL corresponds to the difference of samples' weights determined before and after cooking [6]:

(1) $$\begin{array}{c} \text{CL}=\left[\left(\frac{\text{raw meat weight }-\text{ cooked meat weight}}{\text{raw meat weight}}\right)\right]*100. \end{array}$$

Water-holding capacity (WHC) was based on the percentage of free water in the meat. The samples were sliced into 1 cm thick steaks, with a diameter of 4 cm^2^, wrapped in a layer of gauze, and placed between 18 preweighed WATTMAN papers. The WHC was calculated as the difference in sample weight before and after applying a mechanical force of 2.250 kg for 5 min in accordance with the method described by Grau and Hamm [7]:

(2) $$\begin{array}{c} \text{WHC}\left(\%\right)=\left[\left(\frac{\text{initial weight of the meat }-\text{ final weight of the meat}}{\text{initial weight of the meat}}\right)\right]*100. \end{array}$$

### 2.3. Chemical Analyses

Before analyses, the *longissimus lumborum* muscle (LLM) samples were frozen, lyophilized, crushed, and stored at −20°C for subsequent analyses.

### 2.4. Proximate Composition

Dry matter was calculated using the drying method in a stove for 100°C ± 3°C [8]. Intramuscular fat (IMF) was extracted and quantified using a mixture of chloroform/methanol/water (2/1/1; v/v/v) according to the method described by Bligh and Dyer [9]. Ash was determined by incineration (combustion) in the oven according to AOAC [8]. The total nitrogen was quantified using the Kjeldahl method according to AOAC [8]. Total protein was calculated using the conversion index of 6.25.

### 2.5. Amino Acid Composition

A 300 mg of the lyophilized meat powder sample was dissolved in 10 ml of 6 N HCl, containing 0.1% phenol, followed by hydrolyzation under nitrogen at 110°C for 24 h. Afterward, the pH was adjusted to 2.2 with 7.5 N of NaOH. 0.5 ml of the internal standard (norleucine 50 M) was added, and then the samples were diluted with citrate buffer. Finally, the solution was filtered. A 20 L aliquot of the filtrate was analyzed using a high-performance liquid chromatography (Biochrom 20 Plus amino acid analyzer, Pharmacia, Cambridge, UK), equipped with sodium-oxidized column, cation-exchange resin, and postcolumn derivatization of the amino acids to ninhydrin and spectrophotometric detection at 570 nm, except for proline, which was detected at 440 nm. It should be noted that, in this study, the hydrolysis performed in the amino acids' experimental protocol is not specific for determining the presence and quantification of tryptophan.

The chemical index (CI) corresponded to the minimum ratio between the percentages of each essential amino acid (EAA) of meat protein, compared to each of the corresponding EAA present in the reference protein. Protein digestibility-corrected amino acid score (PDCAAS) was determined, according to FAO/WHO/UNU [10], where PDCAAS = CI × true digestibility of the meat.

The essential amino acids index (IEAA) was also calculated by applying the following equation described by Hepher [11]:

(3) $$\begin{array}{c} \sqrt[{n}]{\frac{a}{ap}*\frac{b}{bp}*\frac{c}{cp}*\ldots \frac{j}{jp}}, \end{array}$$

where *a*, *b*, *c*,…*j* is the content of EAA in each sample; *ap*, *bp*, *cp*,…,  *jp* is the content of EAA in protein standard [10]; and *n* is the number of amino acids used.

The chemical score of the EAA (CSEAA) was calculated according to the reference protein proposed by FAO/WHO/UNU [10] applying the following equation:

(4) $$\begin{array}{c} \text{CSEAA}=\left[\frac{g\text{ EAA in tested protein}}{g\;\left(\text{EAA in pattern protein}\right)}\right]*100. \end{array}$$

### 2.6. Mineral Element

The studied mineral elements were determined after mineralization by atomic absorption spectrometry (Perkin Elmer Atomic Absorption Spectrometer Analyst 200) according to the method described in the International Standard Organization (ISO) number 6869 : 2000, except for phosphorus which had been measured by a spectrophotometer UV visible at 700 nm the Shimadzu type UV-1205, method ISO number 6491 : 1998 [12]. The quantified minerals are phosphorous, magnesium, potassium, calcium, sodium, copper, zinc, iron, and selenium.

### 2.7. Statistical Analysis

Experimental results were presented as means ± standard deviation of triplicate determinations for each sample and each parameter. The statistical analyses were achieved using the software SPSS version 20. The Shapiro–Wilk test was used to verify the samples normal distribution. One-way ANOVA statistical analysis and Tukey's post hoc test were used for means comparison between studied sheep meats; the difference was considered significant (at *p* < 0.05). Principal component analysis (PCA) was performed on the physicochemical dataset to verify whether it was possible to differentiate the samples according to their meat quality characteristics and to obtain more information on the variables that mainly influence the meat quality.

## 3. Result and Discussion

### 3.1. Meat Quality Parameters

The meat parameter quality results are presented in Table 1. pH was closely related to animal and carcass stress conditions in pre- and postslaughter, respectively [13]. The obtained ultimate pH (pH_24_) values ranging between 5.73 and 5.78 indicate that animals were not stressed at slaughter and translate the normal *postmortem* glycolysis. No significant differences were observed between breeds (*p* > 0.05). The recorded ultimate pH was within the normal ranges for commercial meats [14], which were similar to those reported for lambs of different breeds in other studies [15–17]. The pH evolution in postmortem is an essential determinant of organoleptic meat quality (color, juiciness, and tenderness), because it has a direct effect on meat functional proprieties, closely associated with meat biochemical process evolved after slaughter [18].

The meat color is the first organoleptic quality perceived before meat purchase; it is the meat's commercial quality, in other words, the seller aspect of meat [19, 20]. The obtained results show that the meat's color parameters particularly lightness (*L*^*∗*^, *p* < 0.001) and hue (*H*^*∗*^, *p* < 0.01) were influenced by breed and geographical area (*p* < 0.05). The recorded values of lightness ranged from 35.71 to 45.33 which is an indicator of meat with a bright red color sought in the meat industry [20]. The muscles of BGA (*L*^*∗*^ = 45) and ODj breeds (*L*^*∗*^ = 41) were bright and redder than that of Ti (*L*^*∗*^ = 36)) and BGT (*L*^*∗*^ = 35) breeds. The Ti (*a*^*∗*^ = 14.13; *H*^*∗*^: 45.30) and ODj (*a*^*∗*^ = 14.39: *H*^*∗*^: 42.56) muscles were yellower and orange than the muscle of BG breed. This result could be explained by various parameters such as intramuscular fat (IMF), the physical structure of muscle proteins, age of animals, diet, myoglobin concentration, and chemical state of myoglobin [5]. Although animals were at the same age at slaughter, there were no significant differences in the IMF and ultimate pH. Therefore, the difference in meat color between the studied meats could be due to the diet (the antioxidant activity of the meat). Compared with other studies [14, 21, 22], the BG, Ti, and ODj breeds have the highest values of *C*^*∗*^ (18.81, 17.7, 19.9, and 19.89, respectively, for BGA, BGT, Ti and ODj); as a consequence, higher haem pigment content, which could be attributed to enforced exercise in the pastoral production system, was related to transhumance (long-distance covered between winter and summer pastures). However, similar values of *C*^*∗*^ were reported by Budimir, Trombetta [16] in meat for Bergamasca light lambs.

The meat juiciness is an organoleptic quality perceived during chewing. It is strongly influenced by pH evolution in postmortem and by IMF [23]. In the present study, the juiciness of the meat was estimated by two methods. One of them was application of a mechanical force (water-holding capacity) and the other was thermal (cooking loss). The results showed that the geographical area had a nonsignificant effect on WHC and CL (*p* > 0.05), while the Ti breed had lower CL value (*p* < 0.05) than other studied meats. The recorded average values of water-holding capacity ranged between 20.56% for ODj and 26.50% for the BGT breed (*p* < 0.05). The higher value of cooking loss was recorded in meat of BGT breed (38.79%; *p* < 0.05). The high water retention values in both treatments were recorded for Ti lambs, while the lower was recorded for BG. This result indicated that BG meat loses its juice easily in both treatments. Therefore, for the taste (organoleptic) and technologic plan (experiments), we could deduce that BG meat would be more tender (rapid denaturation of myofibrillar structure which allows the intramuscular water to flow) and more stable (low water activity) than Ti and ODj meats. The obtained result of CL for BG lambs was higher than those reported by Yousefi et al. [22] for Chall and Zel lambs, while the lower values were presented by Ti and ODj lambs.

### 3.2. Proximate Composition

As shown in Table 2, we noted that the proximate composition is not affected by breed and geographical area (*p* > 0.05). The dry matter varied slightly (26.15–27.13%). This result is in agreement with a Spanish study on three lamb genotypes [24] where breed did not show a significant effect on dry matter (*p* > 0.05). Our results were higher than those reported by Liu et al. [25] and Berrighi et al. [26], respectively, for Chinese (23–25%) and Algeria lambs (18%). The total meat protein varied between 19.95 for ODj and 22.18 for BGT. The obtained results were comparable to those reported by Addis et al. [15] for Agnello di Sardegna lambs, while they were lower than those reported by Liu et al. [25] for Oula lambs. Finally, mineral matter ranged between 0.71 for BGA and 1.06 for Ti lambs. This result was lower than that reported by Liu et al. [25] and Berrighi et al. [26] in lamb meat.

### 3.3. Mineral Content

After protein and fat, meat is an essential source of several other nutrients, such as minerals [27]. It is defined as poor food in calcium and rich in potassium (K), phosphorus (P), iron (Fe), sodium (Na), and zinc (Zn) [28]. Its consumption can respond qualitatively or quantitatively to the human needs for mineral elements. The estimation of mineral content in *longissimus lumborum* of the studied meat is presented in Table 3. The results showed that the genotype strongly influences the mineral content of the analyzed meats, which present mineral contents ranging (expressed as an mg/100 g of fresh meat) from 976.89 mg in BGA meat to 1072.84 mg in BGT meat. Fe and Zn were most abundant microelements, while selenium was the least abundant (Table 3). Kasap et al. [29] had obtained similar results in the meat produced by Croatian suckling lambs produced extensively, in which the most represented microelements were Fe and Zn. The analyzed meats had a micromineral content (expressed as an mg/100 g fresh meat) which varies between 5.36 mg/100 g in ODj and 5.7 mg/100 g in Ti lamb meat. Except for Fe, all identified microelements were influenced by breed effect without having any effect with regard to geographical area (*p* > 0.05). Meat is a major source of total iron (Fe) and heme iron [30] and thus might help reduce major public health problem worldwide [28, 31]. The iron content depends on the breed, muscle, and animal's age [24]. The present study showed no difference in Fe content between the studied meats (*p* > 0.05). BG, Ti, and ODj meats contained a higher Fe level (2.60 to 2.93 mg/100 g FM) compared to other previous published works, for instance, the works of Van Heerden et al. [32] in South Africa lambs, De Freitas et al. [33] in meat of Braford and Herdford (1.13 to 1.52 mg/100 g FM) in southern Brazil, and Kasap et al. [29] in the three Croatian sheep breed meats (1.90 to 2.02 mg/100 FM). These recorded differences could be explained by pedological reasons such as the alkalinity of soils [34], which influenced iron availability. The Fe content in alkaline soil (pH > 6) was scarcely available for grass [35]. Note that the soils of the eastern region of Morocco and the Middle Atlas were alkaline soils [36], which are rich in iron and could be the source of this recorded richness in iron in studied meats.

The meat zinc is better absorbed by the body than that provided by other foods. Red meat is one of the best dietary sources of zinc with both high levels and very good availability [28]. The copper and zinc content showed a significant breed effect (*p* < 0.001). The higher values were recorded in BGT sheep meat (0.11 mg and 2.78 mg/100 FM for Cu and Zn, respectively). Our results were greater than those found in the meat of Cres et al. [29]. For Cu, the results were lower than those found in the meat of Krk lamb [29]. This finding could be attributed to genotype for Cu [37] and soil-grass for Zn [31]. Kelman et al. [38] had reported a negative correlation between the lean capacity of the meat and the minerals content, in particular of the iron and zinc. Previous studies had shown that the selection of animals for lean meat production (less fat) leads to increases in the proportion of muscles with lower oxidative capacity, consequently lower concentration of myoglobin, less vascularization, and fewer mitochondria (lower Fe and Zn concentrations). This obtained result could be related to extensive feeding adopted by Moroccan breeders, and with pastoralism practice (intense physical activity which will lead to a high vascularity and therefore a richness in iron and zinc) which characterized sheep farming in Morocco, in direct relation with transhumance [39, 40].

In the present investigation, Se content showed a significant effect of genotype (breed) (*p* < 0.001). The higher value was recorded in ODj meat at 0.021 mg/100 g, which was higher than values reported by Williamson et al. [41] in raw meat in Denmark, United Kingdom, and Australia (0.006 mg, 0.007, and 0.010 mg/100 g, respectively). However, they were lower than the one found in USA (0.30 mg/100 g). Overall, the Se content depends mainly on the individual (age and rearing conditions) and geographical origin [42]. These differences could be explained mainly by soil reasons and by the food calendar adopted in Morocco dominated by grazing. Hintze et al. [43] and Hintze et al. [42] had shown that the Se concentration is mainly determined by geographical origins, and the concentration of Se in plants generally reflects its content and availability in soils. Thus, Cabrera et al. [44] reported that meats from pasture were rich in Se.

Concerning the macroelements, the results are shown in Table 3. The macroelement summed up the range (expressed as an mg/100 g of fresh meat) between 971.24 mg/100 g in BGA and 1067.3 mg/100 g in BGT meat. Potassium and phosphorus were the two most abundant minerals, followed by sodium, calcium, and magnesium. The obtained results were higher than those reported by Osorio et al. [45] in suckling lambs of Churra breed and by Polidori et al. [21] for Fabrianese breed meat. All macroelements were influenced by breed effect (*p* < 0.05). In comparison with the literature, the tested meats presented a high content of major mineral elements [21, 29, 45]. The natural feeding condition-based pasture in phosphocalcic soils rich in phosphorus, sodium, magnesium, and calcium could explain the high levels recorded in the studied meats [36] and with free access to the mineral-rich licking block.

The calcium content was significantly influenced by breed (*p* > 0.001). The higher values of calcium and magnesium were recorded in Ti meat, while the highest values of potassium and phosphorous were found in BG sheep meat. This difference could be explained by the dominance of halophilic and xerophilic plants in the eastern region of Morocco rich in sodium and P [46] such as *Atriplex halimus*, *Salsola vermiculata*, *Stipa tenacissima,* and *Artemisia herba-alba* [47]. Except for Mg^2+^ and Ca^2+^, all macroelement contents were affected by geographical area (*p* < 0.05). This result might be due to the decreasing bioclimatic gradient from North to South, ranging from semiarid to arid-lower and pre-Saharan which is correlated with the alkalization of the soil and therefore by the mineral salt content [36].

### 3.4. Amino Acid Content

Meat is a necessary source of EAA for a healthy and balanced diet. Amino acids (AAs) play an essential role in the assessment of meat nutritional value. Thus, they are extremely involved in sensory qualitative determination, by the formation of precursors responsible for taste and flavor during cooking via Maillard reactions. It should be noted that, in this study, the hydrolysis performed in the amino acids' experimental protocol is not specific for determining the presence and quantification of tryptophan. The obtained results of amino acid composition determined in *longissimus lumborum* muscles taken from BG, Ti, and ODj lambs are shown in Table 4. The glutamic and aspartic acid showed the highest values in all samples (from 9.03 to 9.62 for glutamic acid and 5.95–6.40 g/100 g of dry matter of meat for aspartic acid for BGA and ODj, respectively), while the lowest values were found in cysteine (from 0.29 to 0.66%) followed by methionine (1.82–2.34). In the fraction of essential amino acids (EAA), leucine and lysine were the most abundant ones, followed by threonine. From a qualitative point of view, the four obtained amino acid profiles were in consistent with that reported by Belhaj et al. [48] in the samples of Beni-Guil sheep meat collected in March 2016. However, from a quantitative point of view, they were higher than those reported by Belhaj et al. (2018). This difference is probably due to the grazing abundance since the eastern region of Morocco experienced significant rainfall in 2017 than in 2016. Moreover, the results showed that genotype has strongly influenced the meat protein value. The analyzed samples show (i) richness in amino acids (expressed in % of dry matter) which varies between a minimum of 59.79% in BGA and a maximum of 64.77% in Ti meat and (ii) protein values with an essential amino acid index (IEAA) which varies between 158.71 for BGA and 162.20 for Ti meat. The highest values of protein digestibility-corrected amino acid score (PDCAAS) were recorded in Ti (325.09) and ODj (308.78) sheep meats. This may be due to the forest-based pasture system, which was characterized by a diet that mainly includes the oak corns rich in the nitrogenous matter. Several studies have already shown the beneficial effect of oak corns on the nutritional value of meat [49].

The mean values of the CSEAA (CS) are shown in Table 5. The results showed that there is no limiting (restrictive) amino acid in meats analyzed. All the studied meats were rich in EAA necessary for the proper functioning of the human body (Table 5). Statistically, BG and ODj meats had similar protein values (IEAA and PDCAAS), slightly less than Ti meat (Tables 4 and 5). This result could be explained by the livestock system since the BG and ODj sheep were reared in the same cradle, same livestock system, and practically same feeding. In comparison with the reported results by Polidori et al. [21] in the meat of Fabrianese sheep breed and Przybylski et al. [50] in the meat of Corriedale lambs, the studied meats had a high protein quality and are rich in essential amino acids. The nature of the breeding system adopted by Moroccan breeders was based mainly on pasture rich in greenery (rich in nitrogenous materials) such as *Artemisia herba-alba*, *Stipa tenacissima,* and *Atriplex halimus*, which can explain this recorded wealth [51–53].

### 3.5. Principal Component Analysis of the Meat Quality Traits

**Figure 1:**
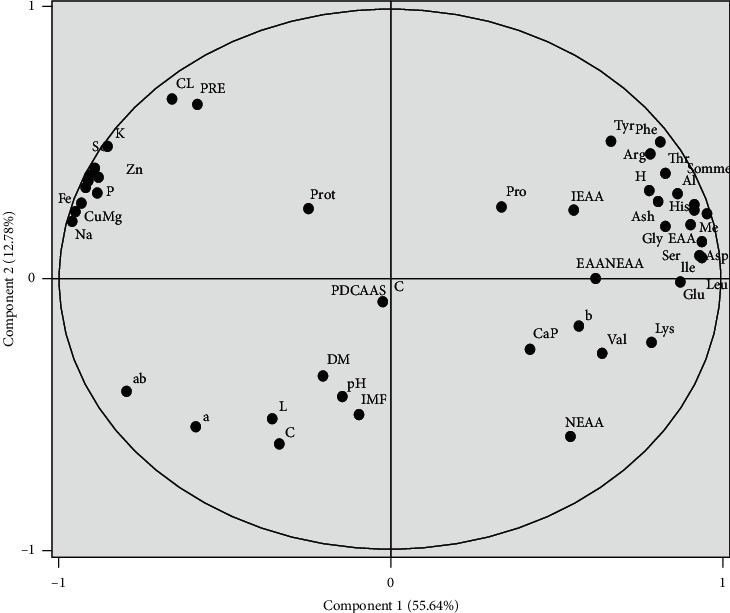
Projection of meat constituents and quality characteristics parameters in the plane defined by two principal components.

**Figure 2:**
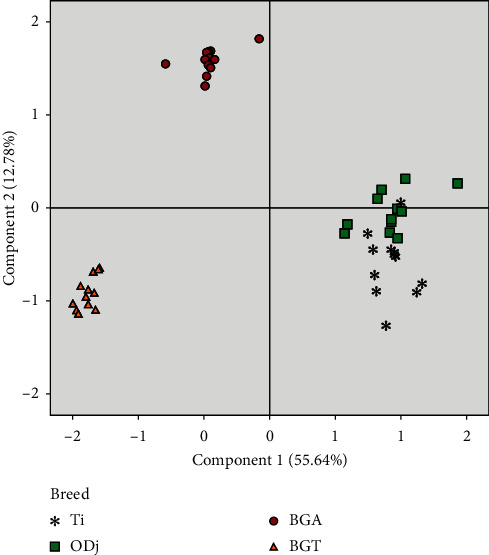
Projection of the variables of the four female lamb groups studied in the plane defined by two principal components.

Principal component analysis (PCA) was carried out to provide a simple visualization of the relationships among proximate composition (dry matter, ash, intramuscular fat, and total protein), meat organoleptic characteristics (meat color, cooking loss, water-holding capacity, and ultimate pH), and minerals and amino acids of four sheep meats produced in Morocco (Figures 1 and 2). The results of this analysis indicate that the majority of the evaluated parameters were influenced by the genotype effect and geographical area. The PCA makes it possible to visualize a large number of variables on a single graph and to estimate the statistical links existing between the studied individuals. This analysis highlighted six main components explaining 90% of the total information. The first and second components (PC1 and PC2) accounted for 55.64 and 12.78% of the total variation, respectively. Figures 1 and 2 provide the global representation of studied animals in the factorial plane consisting of PC1 and PC2. The individual projections on the factorial map showed discrimination between the studied animals making it possible to summarize the interpretations already mentioned above in a very simplified way. Considering PC1, Ti, and ODj breeds were located on the right side of the Figure 2 and were differentiated from BG breed. These results showed that the existence of discrimination between the animals according to their meat quality characteristics. Ti and ODj were correlated with hue and essential amino acids parameters, indicating that these breeds had meat with higher *a*^*∗*^ value and rich in essential amino acids. BG lambs were differentiated from the other lamb types and were located on the left side of Figure 2, where minerals and *a*/*b* ratio were located, showing that animals of BG breed had a bright red color of meat and richness of minerals. For PC2, the most distinct variables were amino acid and mineral contents. According to PC2, the region effect had mainly manifested between animals of the Beni-Guil breed. As shown in Figure 2, the animals of BG breed sampled in the Ain Beni Methar region (BGA) were opposite to those sampled in the Tendrara region (BGT). The animals of BGT were characterized by a higher red color of meat and low values retention water capacity (Figures 1 and 2).

The results of this statistical analysis allowed us to highlight a clear variability between studied animals according to data on meat quality characteristics. This was in agreement with another research that has been carried out on other lambs and revealed the same effects from different geographical areas.

## 4. Conclusion

Livestock production system as a whole plays a major role in the healthiness profile of sheep meat, resulting in significant improvements in terms of amino acids and minerals content, in lambs raised on a pastoral system. Sheep meats from pastoral systems have valuable and essential nutrients necessary to a balanced and complete diet. Essential amino acids and minerals, particularly microelements, are largely present in sampled meat, covering the requirements for humans. This study provided new insights on organoleptic and nutritional value of three Moroccan sheep meats (Beni-Guil, Timahdite, and Ouled-Djellal sheep) reared in the outdoor production system. From a nutritional point of view, the studied meats have a good protein and mineral quality, which is due to their richness in EAA and trace element. The majority of the evaluated parameters were affected by genetic and environmental factors. It is increasingly necessary to value meat produced in the pastures of Morocco on the international meat market. Therefore, the practice of pastoralism must be encouraged and valued. It will have benefits both for Moroccan breeders and for global consumers.

This study confirms that sheep meat produced in natural pasture or reared outdoor provide significant nutritional values that should be encouraged to value Moroccan sheep meat in the international market.

## Figures and Tables

**Table 1:** Meat characteristics of Beni-Guil, Timahdite, and Ouled-Djellal sheep meats.

Breed					
Parameters	BGA	BGT	Ti	ODj	Effect
Ultimate pH	5.78 ± 0.05	5.73 ± 0.05	5.77 ± 0.06	5.75 ± 0.06	NS
CL (%)	34.65 ± 2.74^abd^	38.79 ± 2.22^ab^	29.17 ± 2.52^cd^	31.78 ± 2.79^acd^	^*∗*^
WHC (%)	25.10 ± 1.06^a^	26.50 ± 1.45^a^	21.72 ± 2.05^b^	20.56 ± 3.03^b^	^*∗∗∗*^

*Meat color*					
*L* ^*∗*^	45.33 ± 0.97^a^	35.71 ± 1.75^b^	36.27 ± 3.19^b^	41.69 ± 5.21^a^	^*∗∗∗*^
*a* ^*∗*^	15.38 ± 1.62^a^	13.58 ± 1.29^ab^	14.13 ± 3.86^b^	14.39 ± 2.11^b^	^*∗*^
*b* ^*∗*^	10.84 ± 0.92^a^	11.35 ± 1.03^a^	13.87 ± 1.45^b^	13.55 ± 3.69^b^	^*∗*^
*a* ^*∗*^/*b*^*∗*^	1.45 ± 0.32^a^	1.20 ± 0.05^a^	1.01 ± 0.21^b^	1.13 ± 0.30^b^	^*∗∗∗*^
Chromaticity	18.81 ± 3.50^a^	17.70 ± 1.61^a^	19.90 ± 3.51^b^	19.89 ± 3.55^b^	^*∗∗*^
Hue angle°	35.17 ± 4.50^a^	39.91 ± 1.21^ab^	45.30 ± 2.51^b^	42.56 ± 2.08^ab^	^*∗∗*^

**N**S: not significant; ^*∗*^*p* < 0.05; ^*∗∗*^*p* < 0.01; ^*∗∗∗*^*p* < 0.01;^a,b,c,d^ values with different letters are significantly different within rows; CL: cooking loss; WHC: water-holding capacity; *L*^*∗*^: lightness; *a*^*∗*^: redness; *b*^*∗*^: yellowness; chromaticity: 0–60; hue: 0–360; BGA: female lambs of Beni-Guil breed sampled in the Ain Beni Methar region; BGT: female lambs of Beni-Guil breed sampled in the Tendrera region; Ti: Timahdite breed; ODj: Ouled-Djellal breed.

**Table 2:** Proximate composition of Beni-Guil, Timahdite, and Ouled-Djellal sheep meats.

Breed					
Parameters (%)	BGA	BGT	Ti	ODj	Effect
Dry matter	27.13 ± 1.34	26.15 ± 0.94	26.59 ± 1.27	26.37 ± 0.76	NS
Intramuscular fat	5.14 ± 1.31	4.80 ± 1.00	5.50 ± 1.30	4.60 ± 0.88	NS
Total protein	21.02 ± 3.08	22.18 ± 2.41	20.22 ± 2.41	19.95 ± 1.35	NS
Ash	0.71 ± 0.15^a^	0.97 ± 0.17 ^ab^	1.06 ± 0.14^b^	1.05 ± 0.02^b^	^*∗∗*^

**NS**: not significant; ^*∗*^*p* < 0.05; ^*∗∗*^*p* < 0.01; ^*∗∗∗*^*p* < 0.001; ^a,b^ values with different letters are significantly (*p* < 0.05) different within rows; BGA: female lambs of Beni-Guil breed sampled in the Ain Beni Methar region; BGT: female lambs of Beni-Guil breed sampled in the Tendrera region; Ti: Timahdite breed; OD**j**: Ouled-Djellal breed.

**Table 3:** Minerals composition of Beni-Guil, Timahdite, and Ouled-Djellal sheep meats.

Breed					
Mineral element (mg/100 g FM)	BGA	BGT	Ti	ODj	Effect
Copper	0.11 ± 0.01^a^	0.11 ± 0.01^ac^	0.09 ± 0.01^b^	0.10 ± 0.01^bc^	^*∗∗∗*^
Zinc	2.67 ± 0.13^a^	2.78 ± 0.21^a^	2.66 ± 0.17^a^	2.38 ± 0.05^b^	^*∗∗*^
Iron	2.84 ± 0.20	2.60 ± 0.19	2.93 ± 0.86	2.86 ± 0.18	NS
Selenium	0.013 ± 0.00^a^	0.013 ± 0.00^a^	0.018 ± 0.00^b^	0.021 ± 0.00^c^	^*∗∗∗*^
**∑ microelement**	**5.64** **±** **0.24**	**5.50** **±** **0.26**	**5.70** **±** **0.86**	**5.36** **±** **0.20**	**NS**
Phosphorous	373.16 ± 10.94^a^	398.47 ± 8.55^b^	369.84 ± 4.42^a^	376.20 ± 4.82^a^	^*∗∗∗*^
Magnesium	38.50 ± 1.26^ab^	39.40 ± 2.^63ab^	39.55 ± 1.96^ab^	35.43 ± 4.70^a^	^*∗∗∗*^
Potassium	388.05 ± 21.44^a^	471.49 ± 13.34^b^	429.86 ± 12.91^c^	435.76 ± 10.29^c^	^*∗∗∗*^
Calcium	43.53 ± 4.93^a^	45.30 ± 4.83^a^	59.00 ± 12.15^b^	41.96 ± 2.54^a^	^*∗∗∗*^
Sodium	129.77 ± 4.12^a^	112.64 ± 5.83^b^	111.08 ± 3.02^b^	113.06 ± 5.06^b^	^*∗∗∗*^
∑ **macroelement**	**971.24** **±** **34.83**^**a**^	**1067.30** **±** **22.07**^**b**^	**1009.32** **±** **18.30**^**c**^	**1002.42** **±** **14.64**^**ac**^	^*∗∗∗*^
Total	976.89 ± 34.93^a^	1072.80 ± 22.08^b^	1015.02 ± 17.84^c^	1007.78 ± 14.69^ac^	^*∗∗∗*^

FM, fresh matter; NS: not significant; ^*∗*^*p* < 0.05; ^*∗∗*^*p* < 0.01; ^*∗∗∗*^*p* < 0.001; ^a,b,c^ values with different letters are significantly (*p* < 0.05) different within rows; BGA: female lambs of Beni-Guil breed sampled in the Ain Beni Methar region; BGT: female lambs of Beni-Guil breed sampled in the Tendrera region; Ti: Timahdite breed; ODj: Ouled-Djellal breed; Total: ∑ microelement + ∑ macroelement.

**Table 4:** Amino acids composition of Beni-Guil, Timahdite, and Ouled-Djellal sheep meats.

Breed					
Amino acids (g/100 g DM)	BGA	BGT	Ti	ODj	Effect
Aspartic acid	5.95 ± 0.05^a^	6.16 ± 0.22^ab^	6.32 ± 0.03^b^	6.40 ± 0.12^b^	^*∗∗*^
Tyrosine	2.80 ± 0.04^a^	2.96 ± 0.06^bc^	2.91 ± 0.09^ab^	3.04 ± 0.04^ab^	^*∗∗∗*^
Serine	3.05 ± 0.03^a^	3.19 ± 0.10^b^	3.23 ± 0.02^b^	3.28 ± 0.05^b^	^*∗∗*^
Glutamic acid	9.03 ± 0.06^a^	9.22 ± 0.37^ab^	9.52 ± 0.16^b^	9.62 ± 0.23^b^	^*∗∗*^
Proline	2.59 ± 0.05	2.75 ± 0.12	2.64 ± 0.56	3.02 ± 0.20	NS
Glycine	3.43 ± 0.04^a^	3.62 ± 0.12^ab^	3.75 ± 0.07^b^	3.64 ± 0.11^ab^	^*∗*^
Alanine	3.91 ± 0.02^a^	4.07 ± 0.15^ab^	4.11 ± 0.02^ab^	4.21 ± 0.09^b^	^*∗∗*^
Cysteine	0.66 ± 0.02^a^	0.65 ± 0.01^a^	0.29 ± 0.10^b^	0.36 ± 0.13^c^	^*∗∗∗*^
Arginine	4.84 ± 0.07^a^	5.12 ± 0.11^b^	5.10 ± 0.12^b^	5.26 ± 0.08^b^	^*∗∗*^
NEAA	**37.08** **±** **1.28**^**ab**^	**35.62** ± 0.39^a^	**37.57** **±** **0.61**^**bc**^	**38.47** **±** **0.72**^**bc**^	^*∗∗*^
Valine	2.43 ± 0.03	2.45 ± 0.10	2.73 ± 0.33	2.56 ± 0.08	NS
Methionine	1.82 ± 0.07^a^	2.15 ± 0.01^b^	2.31 ± 0.07^c^	2.34 ± 0.04^c^	^*∗∗∗*^
Isoleucine	2.97 ± 0.01^a^	3.12 ± 0.05^b^	3.26 ± 0.10^b^	3.21 ± 0.04^b^	^*∗∗∗*^
Leucine	4.31 ± 0.02^a^	4.44 ± 0.04^b^	4.80 ± 0.10^c^	4.67 ± 0.07^bc^	^*∗∗∗*^
Phenylalanine	3.21 ± 0.04^a^	3.42 ± 0.06^b^	3.40 ± 0.05^b^	3.50 ± 0.05^b^	^*∗∗*^
Histidine	1.65 ± 0.29^a^	2.35 ± 0.02^b^	2.63 ± 0.21^b^	2.62 ± 0.15^b^	^*∗∗∗*^
Lysine	3.69 ± 0.01^a^	3.76 ± 0.10^ab^	4.17 ± 0.31^b^	3.94 ± 0.11^ab^	^*∗*^
Threonine	3.42 ± 0.05^a^	3.60 ± 0.10	3.60 ± 0.03^ab^	3.71 ± 0.07^b^	^*∗∗*^
EAA	**24.17** **±** **0.28**^**a**^	**26.05** ± **0.51**^**b**^	**27.20** **±** **1.03**^**b**^	**26.90** **±** **0.52**^**b**^	^*∗∗∗*^
Somme	59.79 ± 0.51	63.13 ± 1.80	64.77 ± 0.44	65.37 ± 1.14	NS
EAA/NEAA	0.68 ± 0.01	0.70 ± 0.01	0.72 ± 0.03	0.70 ± 0.01	NS
CI	296.27 ± 6.52^ab^	282.78 ± 6.94^a^	345.84 ± 42.40^b^	328.46 ± 6.94^ab^	^*∗*^
PDCAAS	278.49 ± 3.73^a^	265.81 ± 6.52^ab^	325.09 ± 39.80^b^	308.78 ± 3.73^ab^	^*∗*^

DM: dry matter; NS: not significant; ^*∗*^*p* < 0.05; ^*∗∗*^*p* < 0.01; ^*∗∗∗*^*p* < 0.001; ^a,b,c^values with different letters are significantly (*p* < 0.05) different within rows; BGA: female lambs of Beni-Guil breed sampled in the Ain Beni Methar region; BGT: female lambs of Beni-Guil breed sampled in the Tendrera region; Ti: Timahdite breed; ODj: Ouled-Djellal breed; EAA: essential amino acids; NEAA: nonessential amino acids; CI: chemical index; PDCAAS: protein digestibility-corrected amino acid score.

**Table 5:** Protein quality index (CS and IEAA) of Beni-Guil, Timahdite and Ouled-Djellal sheep meats.

	Pattern protein	Breed				Effect
		BGA	BGT	Ti	ODj	
CS-valine	3.9	282.71 ± 12.02^a^	296.27 ± 3.97^abc^	345 ± 84±42.41^bc^	328.45 ± 10.55^bc^	^*∗*^
CS-Met-Cys	2.2	573.51 ± 4.36^ab^	536.80 ± 21.93^a^	585.36 ± 18.04^b^	614.92 ± 26.13^b^	^*∗∗*^
CS-isoleucine	3	468.18 ± 7.64^a^	471.12 ± 0.90^a^	536.76 ± 17.64^b^	535.64 ± 6.75^b^	^*∗∗∗*^
CS-threonine	2.3	705.13 ± 20.90^a^	706.91 ± 10.36^a^	773.82 ± 8.03^b^	809.46 ± 15.36^c^	^*∗∗∗*^
CS-leucine	5.9	347.90 ± 3.15^a^	348.11 ± 1.91^a^	402.65 ± 8.51^b^	396.40 ± 6.46^b^	^*∗∗∗*^
CS-Phe-tyr	3.8	757.58 ± 14.99^b^	752.94 ± 11.15^b^	822.03 ± 19.10^b^	861.84 ± 12.91^c^	^*∗∗∗*^
CS-histidine	1.5	707.79 ± 7.46	523.83 ± 92.17	867.74 ± 71.99	542.88 ± 21.34	^*∗*^
CS-lysine	4.5	376.90 ± 10.51a	390.13 ± 1.91^a^	458.66 ± 4.78^b^	438.13 ± 13.01^b^	^*∗∗∗*^
IEAA		158.71 ± 0.43^a^	159.65 ± 0.38^ab^	162.20 ± 0.67^c^	161.02 ± 1.38^bc^	^*∗*^

^*∗*^ *p* < 0.05; ^*∗∗*^*p* < 0.01; ^*∗∗∗*^*p* < 0.001; ^a,b,c^values with different letters are significantly (*p* < 0.05) different within rows; BGA: female lambs of Beni-Guil breed sampled in the Ain Beni Methar region; BGT: female lambs of Beni-Guil breed sampled in the Tendrera region; Ti: Timahdite breed; ODj: Ouled-Djellal breed; pattern protein: reference protein provided by WHO/WHO/UNU (2007); CSEAA or CS: chemical score essential amino acid; IEAA: essential amino acids index; Met-Cys: methionine + cysteine; Phe-Tyr: phenylalanine + tyrosine; values of CS and IEAA are referred only with respect to FAO/WHO/UNU (2007) protein pattern.

## Data Availability

The data used to support the findings of this study are available from the corresponding author upon request.
